# Draft Genome Sequence of *Mycobacterium tuberculosis* KT-0184, Isolated in South Korea

**DOI:** 10.1128/genomeA.01755-15

**Published:** 2016-02-18

**Authors:** Taesoo Kwon, Seung Jung Han, Won Gi Yoo, Mi-ran Yun, Sanghyun Lee, Jong Seok Lee, Dae-Won Kim

**Affiliations:** a School of Biological Sciences, Seoul National University, Seoul, Republic of Korea; b Division of Biosafety Evaluation and Control, Korea National Institute of Health, Korea Centers for Disease Control and Prevention, Chungbuk, Republic of Korea; c Department of Environmental Medical Biology, Chung-Ang University, Seoul, Republic of Korea; d Department of Microbiology and Institute for Immunology and Immunological Diseases, Brain Korea 21 Plus Project for the Medical Sciences, Yonsei University College of Medicine, Seoul, Republic of Korea; e International Tuberculosis Research Center, 236 Gaposunhwan-ro, Masanhappo-gu, Changwon, Republic of Korea

## Abstract

Here, we describe the draft genome sequence of *Mycobacterium tuberculosis* KT-0184, from the Beijing family. This genome will provide insight into the evolution and adaptation of *M. tuberculosis* KT-0184 in human hosts.

## GENOME ANNOUNCEMENT

Tuberculosis (TB) is the leading cause of death worldwide, and almost one-third of the world’s population is infected with *Mycobacterium tuberculosis* (1). In addition, TB from the Beijing family has been found globally and is a major health problem in South Korea (2, 3). Here, we report the draft genomic sequence of *M. tuberculosis* strain KT-0184, which was isolated from a South Korean patient. Based on spoligotyping*, M. tuberculosis* KT-0184 belongs to Beijing family *M. tuberculosis* and was susceptible to first-line anti-TB drugs. *M. tuberculosis* KT-0184 was isolated from the sputum from a patient with active pulmonary TB at Masan National Hospital (MNH) in South Korea. Genomic DNA was isolated from *M. tuberculosis* KT-0184 grown in 7H9 broth (Difco Laboratories, Detroit, MI, USA) supplemented with 10% (vol/vol) oleic acid-albumin-dextrose-catalase (OADC) (Becton, Dickinson, Sparks, MD, USA) for 1 month at 37°C, as previously described.

We constructed a paired-end sequencing library using the Nextera sample preparation kit (Illumina, San Diego, CA, USA). The insert size of the paired-end sequencing library was 500 bp, and the DNA sequencing platform used was Illumina MiSeq. We produced 6,626,240 reads from the whole-genome sequencing, and the coverage was 360.52×. We assembled the reads into 117 contigs with the CLC Genomics Workbench program (version 7.5; CLC bio) (4). The *N*_50_ size of the contigs was 86,782 bp. The KT-0184 strain has a genome of 4,368,202 bp, with 65.6% G+C content. After the contigs were entered into the National Center of Biotechnology Information submission portal, KT-0184 was confirmed to belong to the Beijing family (CCDC5079 RefSeq genome accession no. NC_017523.1 [5]), with 98% sequence identity. We identified 4,099 putative open reading frames (ORFs) with Glimmer (version 3.02) (6), 45 tRNAs with tRNAscan-SE (7), and 3 rRNA genes with RNAmmer (8).

A total of 2,863 genes were assigned to Clusters of Orthologous Groups (COG) functional categories, including 121 genes (4.23%) classified as being involved in cell wall/membrane/envelope biogenesis, and 256 genes (8.94%) classified as being involved in lipid transport and metabolism as the most abundant, except for general function genes (407 genes [14.22%]). This abundance is considered to be related to the fact that *M. tuberculosis* has >100 outer membrane proteins and uses lipids to construct an outer membrane (9). On the other hand, we identified 2,889 single-nucleotide variants (SNVs) and 267 indels with reference to the *M. tuberculosis* H37Rv genome (accession no. NC_000962) with GATK (version 3.2.2) (10). Among the SNVs, 77.6% (2,241) are in ORFs, and the rest are in intergenic regions. Of the indels, 58.5% (93/159) of the insertions and 66.7% (72/108) of the deletions are in ORFs, and the rest are in intergenic regions.

This genome will provide insight into the evolution and adaptation of *M. tuberculosis* KT-0184 in human hosts.

### Nucleotide sequence accession number.

This whole-genome shotgun project has been deposited in DDBJ/EMBL/GenBank under the accession no. JUEX00000000.
